# Characterizing the function of *EPB41L4A* in the predisposition to papillary thyroid carcinoma

**DOI:** 10.1038/s41598-020-76606-0

**Published:** 2020-11-17

**Authors:** Daniel F. Comiskey, Huiling He, Sandya Liyanarachchi, Mehek S. Sheikh, Isabella V. Hendrickson, Lianbo Yu, Pamela L. Brock, Albert de la Chapelle

**Affiliations:** 1 Human Cancer Genetics Program and Department of Cancer Biology and Genetics, Comprehensive Cancer Center, College of Medicine, The Ohio State University Wexner Medical Center, 804 Biomedical Research Tower, 460 W 12th Ave., Columbus, OH 43210 USA; 2 Center for Biostatistics, The Ohio State University Wexner Medical Center, Columbus, OH 43210 USA; 3 Department of Internal Medicine, Comprehensive Cancer Center, The Ohio State University Wexner Medical Center, Columbus, OH 43210 USA

**Keywords:** Genetics, Thyroid cancer

## Abstract

Papillary thyroid carcinoma (PTC) is the most common histotype of thyroid carcinoma. The heritability of PTC is high compared to other cancers, but its underlying causes are unknown. A recent genome-wide association study revealed the association of a variant at the 5q22 locus, rs73227498, with PTC predisposition. We report that rs17134155, a variant in high linkage disequilibrium with rs73227498, is located in an enhancer region downstream of coding transcripts of *EPB41L4A*. Rs17134155 showed significant enhancer activity in luciferase assays, and haplotypes containing the protective allele of this variant conferred a significantly lower risk of PTC. While the index SNP, rs73227498, acted as a significant cis-eQTL for expression of *EPB41L4A*, rs17134155 was a significant cis-sQTL for the alternative splicing of a non-coding transcript of *EPB41L4A*, called *EPB41L4A-203*. We also performed knockdown of *EPB41L4A* followed by microarray analysis. Some of the top differentially-expressed genes were represented among regulators of the WNT/*β*-catenin signaling pathway. Our results indicate that an enhancer region at 5q22 regulates the expression and splicing of *EPB41L4A* transcripts. We also provide evidence that *EPB41L4A* expression is involved in regulating growth and differentiation pathways, suggesting that decreased expression of *EPB41L4A* is a mechanism in the predisposition to PTC.

## Introduction

Thyroid carcinoma is the most common malignancy of the endocrine system^1^. Recent studies of the SEER cancer statistics show that it is among several cancers with the largest increase in standard incidence rates, rising approximately 1.9% each year. Current estimates predict that there will be 52,070 newly-diagnosed cases of thyroid carcinoma in 2020, and 2,170 deaths in the United States^2,3^. This increase of PTC cases seems to be attributable to an increase in neck ultrasounds and the diagnosis of subclinical papillary thyroid microcarcinomas using fine-needle aspiration biopsy^4,5^. Thyroid carcinoma can be broadly divided into medullary (MTC) and non-medullary thyroid carcinoma (NMTC). The vast majority of cases, over 90%, comprise NMTC and are overall less aggressive^6,7^. While most NMTCs are sporadic, a number of case reports and studies have shown the aggregation of non-syndromic thyroid carcinoma in families^1,8–14^.

Thyroid carcinoma has the strongest heritability of all human cancers^15,16^. As the overall somatic mutation burden is low, much of the heritability of thyroid carcinoma is thought to derive from germline variants. In case control studies from Utah and Sweden, first degree relatives of probands had an 8- and 12-fold risk, respectively, to develop follicular or papillary thyroid carcinoma (FTC/PTC)^17,18^. Many linkage studies have disclosed loci and genes predisposing to PTC^8,11,19^. However, the majority of these candidate genes and loci have not disclosed unequivocal pathogenic variants causing PTC. Presently, the largest source of information on the genetics of NMTC are genome-wide association studies (GWASs). To date, there have been six GWASs performed in both Europeans and Asians, with a number of associated loci, including 9q22, 8p12, and 14q13, demonstrating replication in several populations^20–25^. In the past decade, we have collaborated with deCODE to perform GWASs in NMTC and uncovered the association of eleven loci with NTMC and PTC predisposition. Previously, we reported the association of a variant at 5q22, rs73227498, with PTC^22^. This variant is a non-coding variant in close proximity to the gene body for erythrocyte membrane protein band 4.1 like 4A (*EPB41L4A*).

*EPB41L4A* is a member of the band FERM (Four-point-one, Ezrin, Radixin, Moesin) superfamily of proteins. Members of this superfamily comprise a group of membrane-associated proteins whose major biological functions are the regulation of cytoskeletal rearrangements, intracellular trafficking, and WNT/*β*-catenin signaling^26^. Recently, *EPB41L4A* was implicated in the progression of multiple myeloma. High expression of *EPB41L4A* predicted better survival, which was hypothesized as resulting from gene expression changes in genes involved in DNA replication^27^. In addition to coding isoforms of *EPB41L4A*, there are several long non-coding RNA (lncRNA) transcripts, including *EPB41L4A-AS1* and *EPB41L4A-AS2*, both of which have several documented functions in regulating the proliferation and metabolism of cancer cells^27–30^.

The 5q22 locus contains several GWAS signals for a diverse set of phenotypes, including coronary artery disease^31^, mosquito itch^32^, cognitive function^33^, and suicidal behavior^34^. However, the most significant genetic signal at this locus is rs73227498 and its association with PTC^22^. Additionally, the expression of EPB41L4A protein is higher in thyroid compared to other tissues^35^. Taken together, our overall hypothesis is that genetic variants at 5q22 alter the expression of *EPB41L4A* or its non-coding transcripts, leading to changes in gene networks favorable for the development of PTC. Therefore, the aim of our study was to identify the functional variants at this locus, and to investigate the mechanisms by which alterations of *EPB41L4A* contribute to thyroid cancer susceptibility.

## Results

### 5q22 GWAS variant rs73227498 is located downstream of coding *EPB41L4A* transcripts

**Figure 1:**
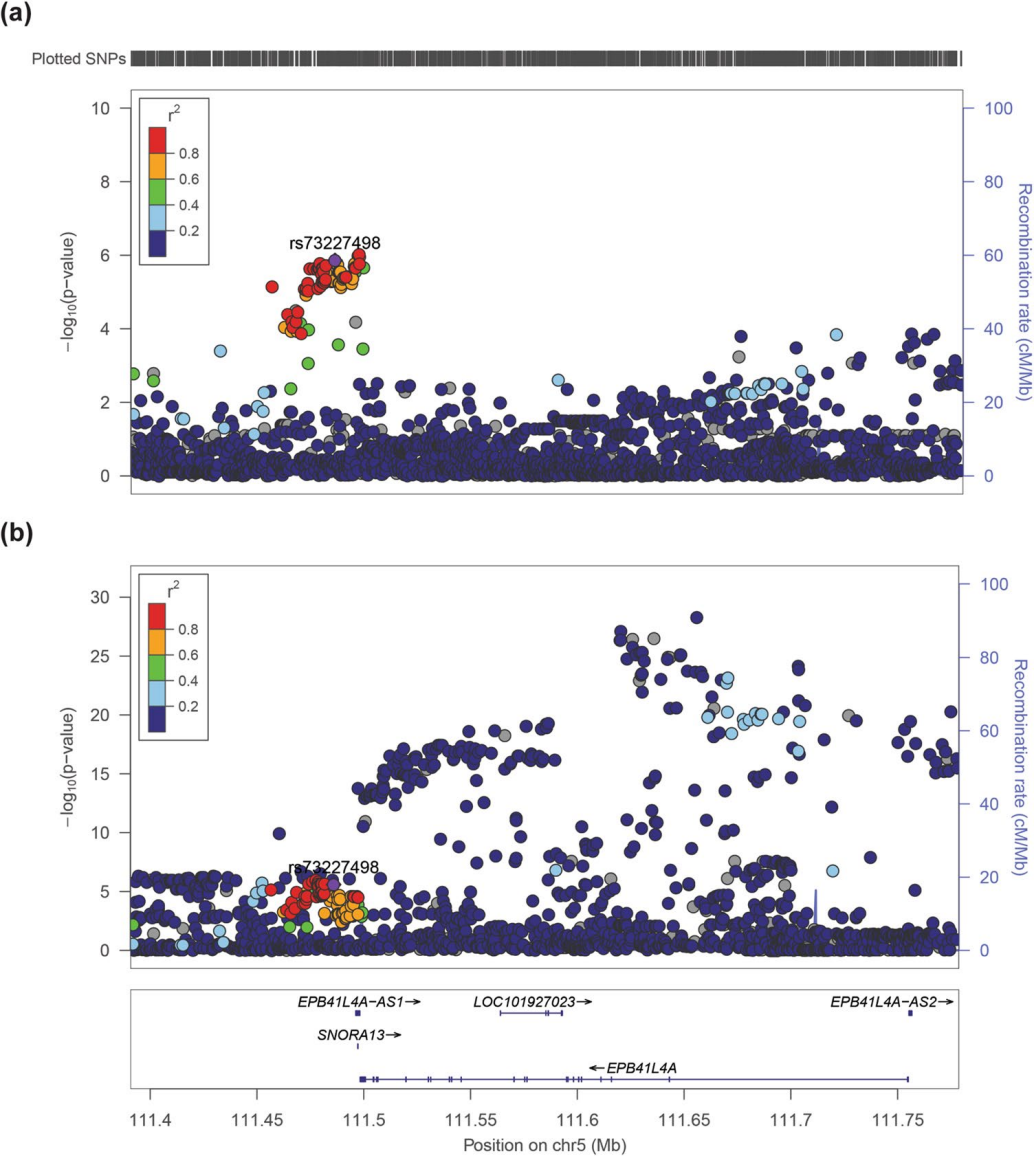
PTC GWAS SNP rs73237498 is located downstream of coding *EPB41L4A* transcripts. (**a**) Graph of negative log_10_-transformed *P* values at the 5q22 locus from Ohio PTC cases and controls in a recent GWAS^22^. (**b**) Graph of negative log_10_-transformed *P* values at the 5q22 locus for expression quantitative trait loci (cis-eQTL) in thyroid tissue.

Our most recent GWAS in PTC identified a variant at 5q22 that was significantly associated with PTC risk; meta-association analysis produced a combined odds ratio of 1.37^22^. The index single-nucleotide polymorphism (SNP), rs73227498, is located 12 kilobases downstream of *EPB41L4A* (Fig. 1a). The transcript level of *EPB41L4A* is highest in thyroid tissue according the GTEx Portal and there are many significant expression quantitative trait loci (cis-eQTL) at this locus (Fig. 1b). However, many of these genotype-expression pairs are located within the gene body and are not in linkage disequilibrium (LD) with rs73227498. To identify other variants that are co-inherited with the index SNP, we performed LD analysis spanning chr5:111390730-111778778. We narrowed down a 20-kilobase region starting with rs73227498 and moving further downstream of *EPB41L4A*. In total, there were 51 variants in LD with rs73227498, which we further narrowed down based on allele frequency, variant annotation, and in silico prediction tools (Supplementary Table S2). We performed haplotype analysis using eleven variants and identified four significant haplotypes: one risk and three protective (Table 1).

**Table 1:** Haplotype analysis of *EPB41L4A* in Ohio PTC cases versus controls.

HapID	Control	Case	OR	*P* value	rs27981	rs76177172	rs73787778	rs79092804	rs79705195	rs78455089	rs56092269	rs76564228	rs17134154	rs17134155	rs732274981
Haplotype 1	0.69	0.74	1.29	1.19E−05	G	C	G	C	G	C	T	A	T	C	A
Haplotype 2	0.07	0.05	0.65	1.02E−04	A	T	C	T	A	A	G	G	C	T	T
Haplotype 3	0.03	0.02	0.66	1.78E−02	A	T	C	T	A	A	G	G	C	C	T
Haplotype 4	0.09	0.07	0.82	4.81E−02	G	C	G	C	G	C	T	A	T	T	A

### SNP rs17134155 is associated with allele-specific transcription

**Figure 2:**
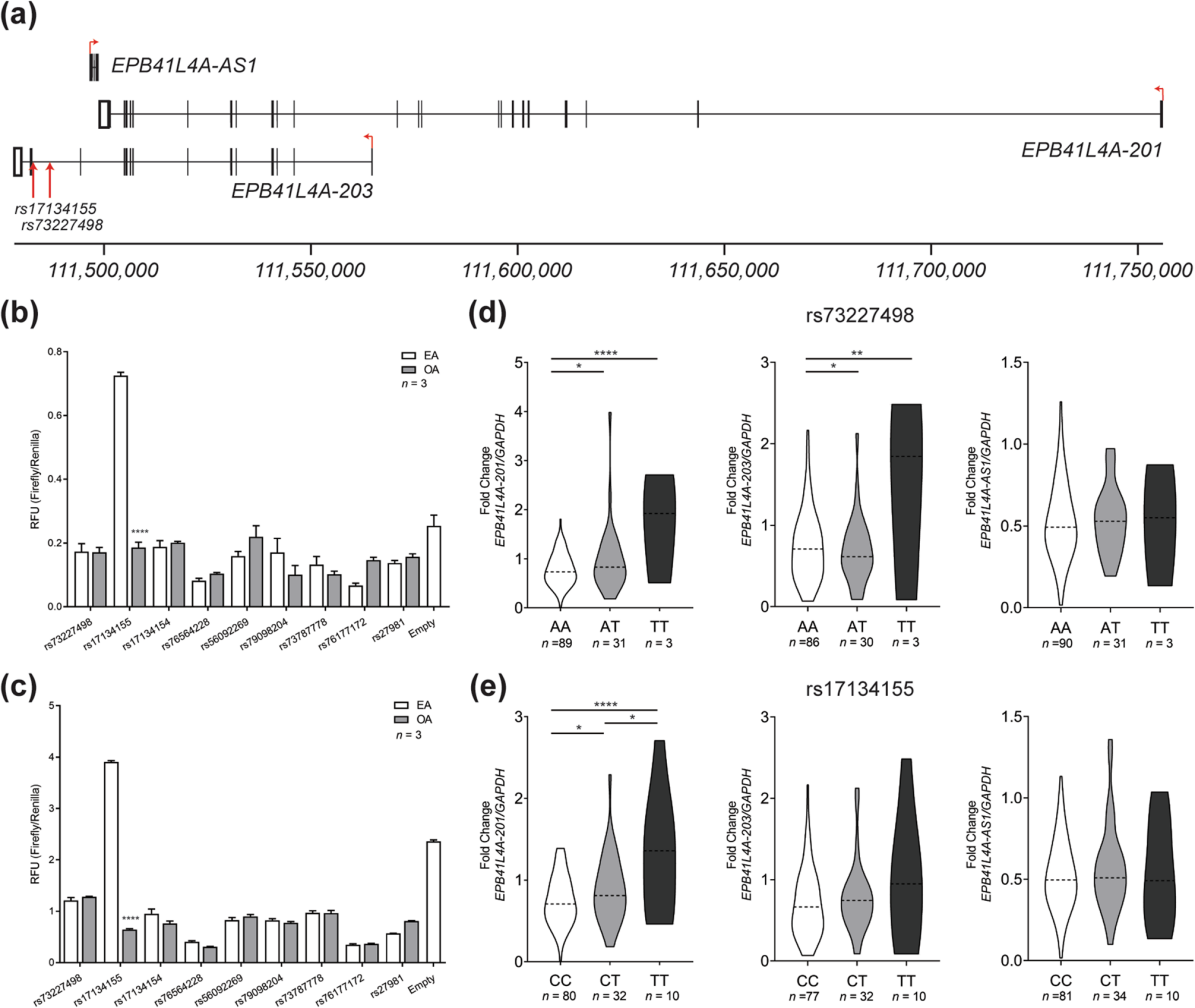
SNP rs17145155 shows allele-specific expression. (**a**) Schematic of coding and non-coding transcripts of *EPB41L4A* and *EPB41L4A-AS1.* The position of GWAS SNP rs73227498 and rs17134155 are indicated by red arrows. Results of dual luciferase activity in relative fluorescence units (RFU) for downstream *EPB41L4A* variants in (**b**) BCPAP and (**c**) TPC1 cells. Results are shown as mean ± SEM of one experiment with four technical replicates. For each variant, the left allele is the effect allele (EA) and the right is the other allele (OA). Asterisks indicate that the experiment was repeated four times with similar results. Allele-specific expression of *EPB41L4A-201*, *EPB41L4A-203*, and *EPB41L4A-AS1* in adjacent unaffected thyroid tissue between genotypes for (**d**) rs73227498 and (**e**) rs17134155. *P < 0.05, **P < 0.01, ****P < 0.0001 (two-tailed *t* test).

The 20-kilobase haplotype block is located within a non-coding transcript of *EPB41L4A*, *EPB41L4A-203* (ENST00000507810.5, Fig. 2a). In order to ascertain the functional importance of these eleven variants, we cloned each variant into an enhancer-specific luciferase vector. We were able to combine three variants into one luciferase construct: rs78455089, rs79705195, and rs79092804. We then transfected the effect allele (EA) and other allele (OA) of each construct into both BCPAP (Fig. 2b) and TPC1 cells (Fig. 2c). In both cell types, rs17134155 showed significant allele-specific expression, with the EA demonstrating enhancer activity. Indeed, rs17134155 is predicted to lie within an enhancer element according to geneHancer (Supplementary Figure S1)^36^. These data are consistent with ChIP-seq data in normal thyroid tissue, which show that this region has enhanced H3K27ac histone marks^37^. Interestingly, the OA of rs17134155 led to a switch from a risk haplotype (Haplotype 1) to a protective haplotype (Haplotype 4; Table 1), providing another line of evidence that this variant has a significant function in regulating PTC risk.

In addition, we performed quantitative polymerase chain reaction (qPCR) for the coding transcript *EPB41L4A-201*, as well as non-coding transcripts *EPB41L4A-203* and *EPB41L4A-AS1* on normal adjacent thyroid tissue samples from PTC patients, which were also genotyped for both rs17134155 and rs73227498. In agreement with the GTEx Portal, we found significant associations between rs73227498 and rs17134155 genotypes and expression of *EPB41L4A* (Fig. 2d,e). Additionally, we found a significant association for rs73227498 genotypes and *EPB41L4A-203* expression (Fig. 2e).

### IPA of *EPB41L4A* knockdown indicates changes in proliferation and differentiation

**Figure 3:**
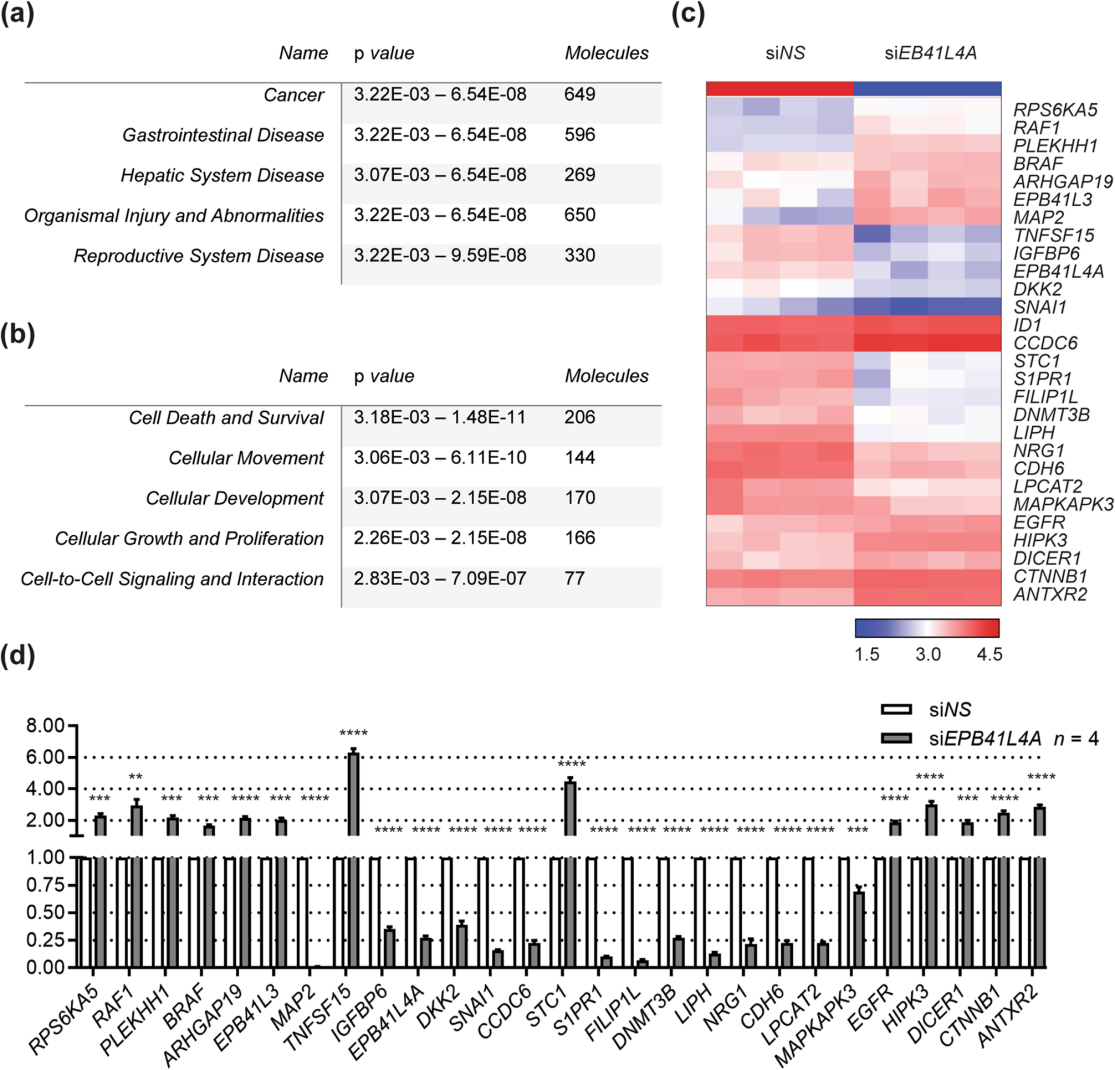
Knockdown of *EPB41L4A* shows changes in cell survival and motility. Ingenuity Pathway Analysis depicting the top (**a**) ‘disease and disorders’ and (**b**) ‘molecular and cellular functions’ affected on *EPB41L4A* knockdown. (**c**) Expression of the candidate genes involved in cellular movement from siRNA knockdown of *EPB41L4A* is plotted with a heat-map color scale using relative expression fold changes (fold change > 2.0, *P* < 0.0001, *n* = 4). (**d**) Quantitative polymerase chain reaction validations for twenty-seven differentially- expressed genes in BCPAP cells treated with non-specific (si*NS*) or *EPB41L4A* (si*EPB41L4A*) siRNA. **P < 0.01, ***P < 0.001, ****P < 0.0001 (two-tailed *t* test).

According to our data, the EA of rs73227498 is associated with lower expression of *EPB41L4A*. Given the role of EPB41L4A in cellular trafficking and *β*-catenin signaling, we decided to interrogate changes in gene expression upon knockdown of *EPB41L4A*. To this end, we transfected BCPAP cells with siRNA against a non-specific target (si*NS*) or *EPB41L4A* (si*EPB41L4A*) for 72 h, hybridized RNA to an Affymetrix microarray, and performed Ingenuity Pathway Analysis. Overall, the most significant change in diseases and disorders was cancer (Fig. 3a), and the most significant changes in molecular and cellular functions were cell proliferation, movement, and development (Fig. 3b). Some of the top differentially-expressed genes were represented among regulators of cell–cell interaction (*FILIP1L*, *MAP2*, *S1PR1*) and cell proliferation/differentiation (*ARHGAP19*, *BRAF*, *EGFR*, *IGFBP6*, *SNAI1*, *TNFSF15*, *MAPKAPK3*). We also observed significant changes in *β*-catenin pathway regulators (*CTNNB1*, *CDH6*, *DKK2*; Fig. 3c). To confirm knockdown of *EPB41L4A* we performed qPCR. We also confirmed changes in gene expression for 26 other differentially-expressed targets that were identified by microarray hybridization (Fig. 3d).

BCPAP cells harbor somatic mutations in *BRAF* and *p53*, a rare combination in the context of PTC. To understand whether some of the same changes in gene expression wrought by *EPB41L4A* knockdown in BCPAP cells were also present in normal thyroid cells, we knocked down *EPB41L4A* in NThy-ori 3–1 cells. We assayed the expression levels of the same 26 target genes and altogether found concordance among 19/26 candidate genes (Supplementary Fig. 2a). The cycle threshold value of *EPB41L4A* after knockdown was undetermined using qPCR, so we demonstrated siRNA knockdown efficiency using a standard RT-PCR (Supplementary Fig. 2b).

### Knockdown of *EPB41L4A* increases thyroid cell viability

**Figure 4:**
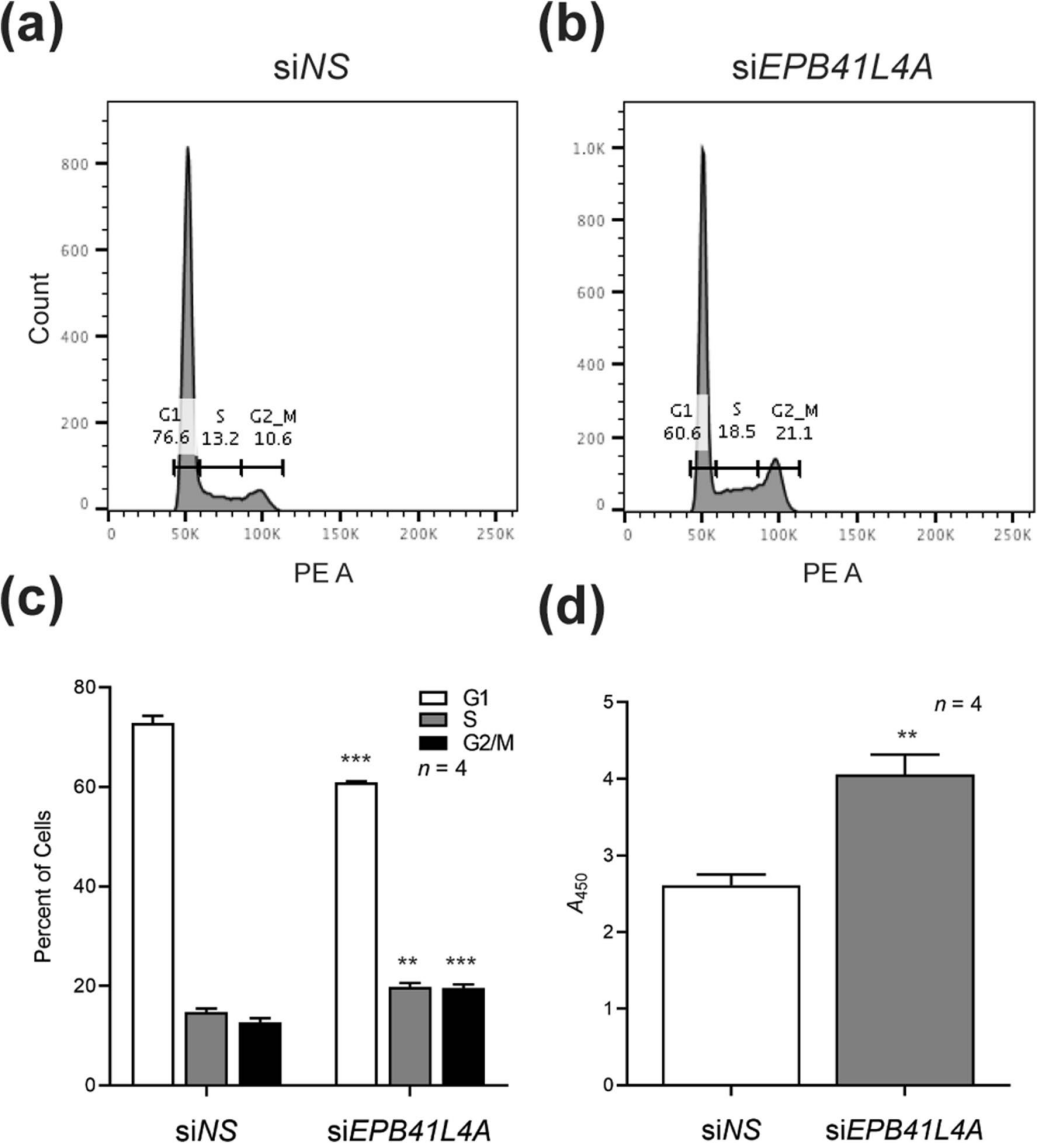
Knockdown of *EPB41L4A* results in increased proliferation of BCPAP cells. Histogram of propidium iodide-stained BCPAP cells treated with (**a**) non-specific siRNA (si*NS*) or (**b**) *EPB41L4A* siRNA (si*EPB41L4A*) for 72 h. The percentage of cells in each phase of the cell cycle (G1, S, G2/M) is shown for each range. (**c**) The average percentage of BCPAP cells in each phase of the cell cycle is shown. (**d**) WST-8 proliferation assay of BCPAP cells. The level of metabolic activity is depicted by the absorbance of formazan dye (*A* = 450). **P < 0.01, ***P < 0.001 (two-tailed *t* test).

The most prominent changes in gene expression we observed upon *EPB41L4A* knockdown were among regulators of cell survival. To test whether *EPB41L4A* knockdown affected viability we performed cell cycle analysis on cells treated with si*NS* (Fig. 4a, Supplementary Fig. 2c) or si*EPB41L4A* (Fig. 4b, Supplementary 2d). We observed a significant decrease in the proportion of both BCPAP and Nthy-ori 3–1 cells in G1 phase and a relative increase of cells in both S and G2/M phases (Fig. 4c, Supplementary Fig. 2d). Additionally, we found that cells treated with si*EPB41L4A* had increased metabolic activity in a WST-8 proliferation assay compared to si*NS* (Fig. 4d, Supplementary Fig. 2e). These data suggest that an increase in cell proliferation due to decreased *EPB41L4A* expression is a possible mechanism in the predisposition to PTC.

### SNP rs17134155 shows allele-specific alternative splicing of *EPB41L4A-203*

**Figure 5:**
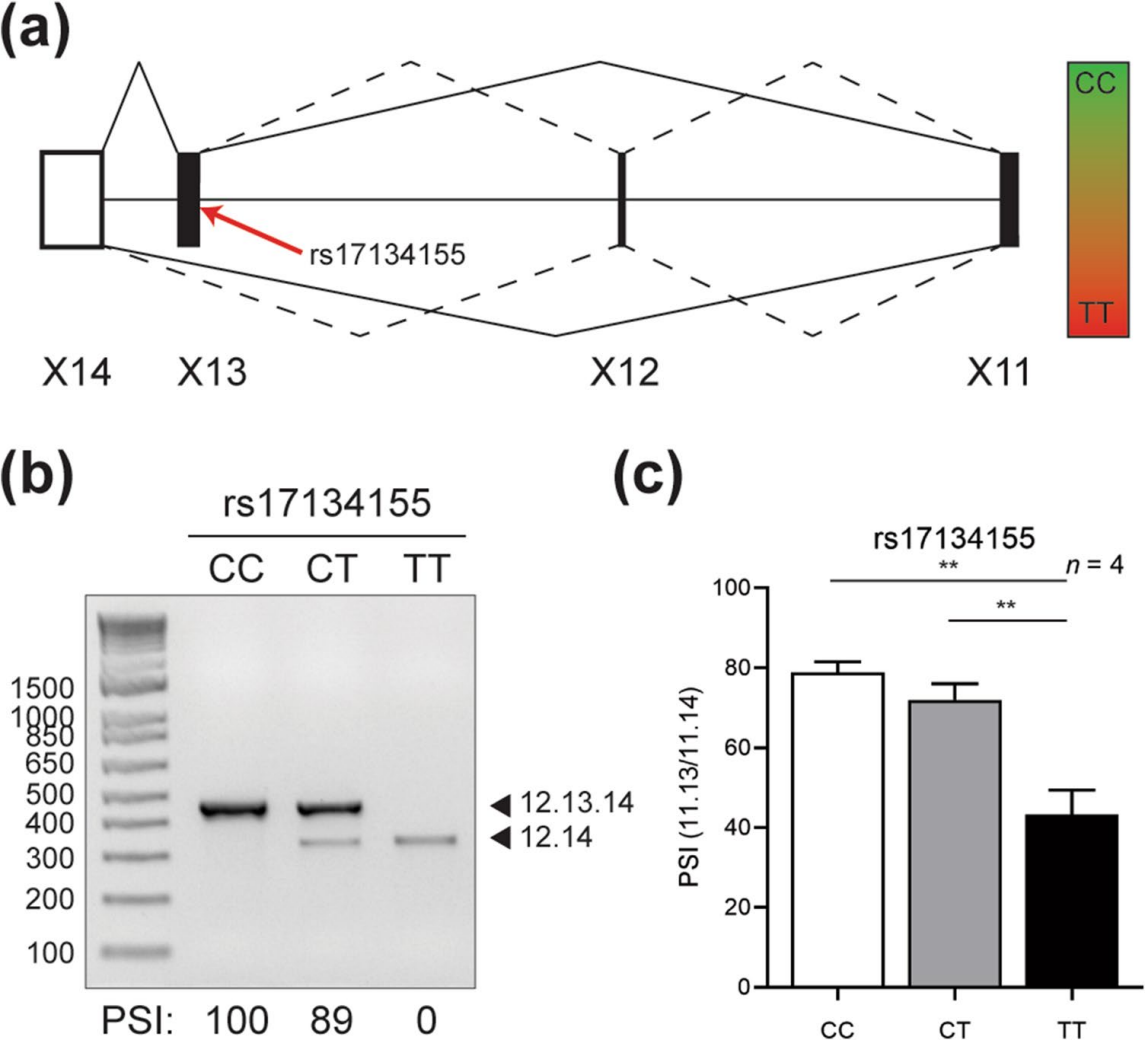
rs17134155 shows changes in alternative splicing of the non-coding *EPB41L4A-203* transcript. (**a**) Schematic of the alternative splicing pattern for *EPB41L4A-203*. Solid lines represent predominant splice forms for rs17134155 “CC” (top) and “TT” (bottom) genotypes. Dotted lines represent lower-expressed splice isoforms. The position of rs17134155 is depicted by a red arrow. (**b**) RT-PCR of *EPB41L4A-203* minor splice products according to rs17134155 genotype. (**c**) Graph of percent splicing inclusion (PSI) for exon-included relative to exon-excluded products for major *EPB41L4A-203* splice variants. **P < 0.01 (two-tailed *t* test).

Rs17134155 is a splice-region variant located at the 3′ splice site of intron 12 of *EPB41L4A-203.* Specifically, the OA of rs17134155 changes the canonical “AG” 3′ splice site to a weak “AA” splice site. This event is predicted to result in a significant change in the alternative splicing of downstream exon 13. In order to validate this prediction, we designed primers in exon 11 and exon 14 of *EPB41L4A-203* and performed an RT-PCR on normal adjacent thyroid tissue from individuals bearing each rs17134155 genotype. In total, we observed three alternative splicing events, resulting in four bands: inclusion of exons 11–14, mutually-exclusive alternative splicing of either exon 12 or 13, or exclusion of both exons 12 and 13. The two most predominant alternative splicing patterns were total exon inclusion or exclusion. There were a minority of transcripts generated from mutually-exclusive alternative splicing of either exon 12 or 13 (Fig. 5a).

We confirmed the alternative splicing of minor splice products using primers in exons 12 and 14. Specifically, we observed the complete inclusion or exclusion of exon 13 on the basis of rs17134155 genotype. The OA of rs17134155 resulted in a reduction of 11 percent splicing inclusion (PSI) in the heterozygous state, whereas the homozygote showed 0% PSI (Fig. 5b). In order to quantitate the levels of alternative splicing between the major alternative splicing transcripts, we designed exon junction primers between exons 11 and 13, as well as exon 11 and 14. We then assessed the relative levels of exon inclusion by comparing the two. Similar to the minor alternative splicing events, we observed that individuals homozygous for the OA of rs17134155 had a significant reduction of PSI in normal adjacent thyroid tissue compared to both “CC” and “CT” genotypes (Fig. 5c).

## Discussion

In the present study we characterized a locus at 5q22 that was reported in a recent GWAS of PTC. We performed LD analysis and identified 51 variants that were in high LD with the index SNP, rs73227498. We narrowed down this list to eleven SNPs for functional validation in a dual luciferase assay. We subsequently identified one SNP, rs17134155, that demonstrated significant differential enhancer activity in TPC1 and BCPAP cell lines. Additionally, both the GWAS index SNP, rs73227498, and rs17134155 had significant cis-eQTL signals for the expression of *EPB41L4A*. To assess the effects of differential transcriptional output from *EPB41L4A*, we knocked it down transiently in BPCAP cells and performed microarray analysis. IPA of our microarray data revealed significant changes in cell death and survival, movement, and differentiation. Changes in thyroid cell viability were confirmed in both cell cycle and WST-8 proliferation assays. Finally, we validated the alternative splicing of a non-coding transcript of *EPB41L4A*, *EPB41LA-203*, in PTC patient samples based on a significant cis-sQTL for rs17134155.

One of the difficulties in characterizing the 5q22 PTC risk locus is its gene annotation in various databases. Originally, rs73227498 was classified as an intergenic variant^22^, but here we described it as an intronic variant. The reason for this is because the gene model for *EPB41L4A* is different between established databases. For example, GENCODE v.32 only supports two transcripts of *EPB41L4A* in its basic gene set, ENST00000261486.6 and ENST00000621003.4, the latter of which is considered a non-coding transcript in both GENCODE^38^ and NCBI RefSeq^39^. However, in ENSEMBL^40^, ENST00000621003.4 is annotated as a coding transcript because of a difference in the reported length of exon 20, in which GENCODE reports the retention of 45 bases of intron 20. The RefSeq database from NCBI lists both transcripts from the GENCODE basic gene set, but includes two more coding isoforms: NM_001347888.2 and NM_001347887.2. NM_001347888.2 is almost identical to ENST00000305368.8 listed in ENSEMBL, however the former is listed as a coding transcript and the latter as a non-coding. Finally, the NM_001347887.2 transcript bears similarity to ENST00000261486.6 at the 5′ end and ENST00000507810.5 on the 3′ end, save for the exclusion of exon 12 of ENST00000507810.5. In summary, the GENCODE basic and RefSeq gene annotations do not agree with what is reported in the ENSEMBL database.

ENSEMBL and the GTEx Portal both list eleven transcripts of *EPB41L4A* in total. While all have GENCODE transcript IDs, only the two GENCODE basic transcripts have transcript support level 1. Both of these transcripts encode identical proteins. The other nine are non-coding transcripts, including *EPB41L4A-203* (ENST00000507810.5). As previously mentioned, this transcript is not included in the RefSeq or GENCODE basic gene set. However, a genuine splice junction between exon 1 and exon 2 (exon 13 of coding transcripts) of this transcript was detected reliably from TaqMan gene expression assays (Fig. 2d,e). Therefore, it is in our estimation that the ENSEMBL and the GTEx Portal have a more complete list of *EPB41L4A* transcripts. It remains to be seen what purpose these other non-coding transcripts serve in regulating the gene expression of coding transcripts or if they have novel functions altogether.

One of the recurring themes of low-penetrance loci in the predisposition to PTC is the presence of lncRNA transcripts that regulate gene expression of a neighboring gene. This features prominently at the 9q22 locus between *PTCSC2* and *FOXE1*^41,42^ and at 14q13 between *PTCSC3* and NKX2-1/MBIP^43^. Regarding 5q22, it is not known whether *EPB41L4A*-203 can directly regulate the expression of *EPB41L4A*. The rs17134155 SNP we characterized lies within a functional enhancer that is also a splice site for *EPB41L4A-203.* We confirmed the effect of this variant on the alternative splicing of *EPB41L4A-20*3 and also its significant association with *EPB41L4A* transcript levels. Analysis of the combined effect of both rs73227498 and rs17134155 on the transcriptional output of *EPB41L4A* and nearby genes should therefore be pursued subsequently.

IPA revealed a significant activation of pathways involved in cell survival upon knockdown of *EPB41L4A*. Since its characterization, EPB41L4A has been recognized as a critical regulator of *β*-catenin and hence, the WNT pathway. Depletion of *β*-catenin from SW480 cells significantly reduced the expression of *EPB41L4A* (*hNBL4*)^26^. *β*-catenin is a regulator of cell proliferation and is also frequently mutated in cancers, including activating mutations in anaplastic thyroid carcinoma^44^. In PTC, several studies have shown the relocalization of *β*-catenin to the cytoplasm, often correlating with increased levels of Cyclin D1^45,46^. While we did not observe the upregulation of Cyclin D1 upon knockdown of *EPB41L4A*, we observed a significant increase in *CTTNB1* (*β*-catenin) expression, as well as a decrease in *DKK2* expression. DKK2 generally acts as a negative regulator of canonical WNT signaling, depending on the cellular context^47^. A related family member, DKK1, has been shown to inhibit thyroid cancer survival^48^ and its downregulation correlates with poorer prognosis in PTC^49^. Furthermore, we also observed significant upregulation of *EGFR* upon knockdown of *EPB41L4A*. Given the evidence for crosstalk between these pathways in the context of PTC^50^, we believe the expression of EPB41L4A may regulate the survival of PTC cells through the WNT/EGFR pathway.

One limitation of this study is that we do not understand what the function of the non-coding *EPB41L4A-203* transcript is or the significance of its alternative splicing. Since most lncRNAs are nuclear, the mature transcript of *EPB41L4A-203* is unlikely to act as a sponge for microRNAs. Furthermore, there is very little sequence homology between the 3′UTR of *EPB41L4A* and any portion of *EPB41L4A-203.* Another difficulty is that since this locus is poorly annotated, this lncRNA does not appear in any databases that predict lncRNA function or RNA-RNA interactions. One hypothesis is that *EPB41L4A-203* could affect the function of *EPB41L4A-AS1*, as its gene body is contained within an intron of *EPB41L4A-203*. In this capacity, the unspliced lncRNA could serve as a sponge for RNA-binding proteins. Still another hypothesis is that the enhancer the rs17134155 SNP lies within is affecting the transcription of *EPB41L4A-AS1*. While we did not observe any changes in *EPB41L4A-AS1* in our genotype-expression analysis, there is some evidence in the GTEx Portal that the protective haplotype leads to an increase in *EPB41L4A-AS1* transcripts in some tissues, such as sun-exposed skin. This would be in line with established reports which find that reduced expression of *EPB41L4A-AS1* is a molecular characteristic of several cancer types^51,52^.

In conclusion, we provide both genetic and biochemical evidence that implicates *EPB41L4A* (OR = 1.37, *P* = 3.0 × 10^−10^) at 5q2*2* in the predisposition to PTC. Our data indicate the presence of an enhancer element downstream of coding transcripts that regulates the levels of *EPB41L4A*, with the protective allele associated with higher *EPB41L4A* expression. This transcriptional control may provide thyroid cancer cells a growth advantage through the EGFR and *β*-catenin signaling pathways. Further studies to uncover the role of lncRNA transcripts derived from *EPB41L4A* will allow for a greater understanding of this risk locus in the context of PTC.

## Methods

### Cell lines

Cell lines were incubated in either DMEM (BCPAP and TPC1) or RPMI (NThy-ori 3-1) medium supplemented with 10% fetal bovine serum, 1X antibiotic–antimycotic, and 1X Plasmocin prophylactic at 37 °C in humidified air with 5% CO_2_. BCPAP and TPC1 cell lines were obtained from Rebecca Schweppe (University of Colorado Cancer Center, Denver, CO). NThy-ori 3-1 cells were obtained from Dr. John Phay (The Ohio State University, OH).

### Generation of plasmid constructs

*EPB41L4A* regulatory variants were PCR-amplified from genomic DNA and cloned into XhoI and EcoRV sites of the pGL4.10-E4TATA vector. All cloning was performed using InFusion HD enzyme.

### Luciferase reporter assay

For the luciferase reporter assay, cells were transiently transfected with reporter plasmids using Lipofectamine 2000 as previously described^53,54^. Each well was transfected with a luciferase reporter plasmid and Renilla plasmid pRL-TK (Promega) as an internal control for each well. Cells were lysed 24 h after transfection with 100 μl passive lysis buffer. An aliquot of cell lysate was assayed for luciferase activity using the GloMax 96 Microplate Luminometer.

### Linkage disequilibrium and haplotype analyses

Linkage disequilibrium (LD) analysis was performed using the genotype data of 503 samples from 1000 Genomes Project (phase 3), European population. Haploview V4.2 software was applied and haplotype blocks were generated using the confidence interval method. 51 variants in LD with the index SNP were filtered such that variants that had a negative transformed-log_10_ *P* value of less than 4.0, a DANN score less than the index SNP (0.57), or those with a DANN score < 0.77, and RegulomeDB score of 6 or 7 were removed. Haplotypes for 11 of the 12 remaining SNPs were generated by using the genotyping and computer imputation data from the European descendants in an Ohio cohort of a recent GWAS^22^. The SHAPEIT V2 program was used to estimate the haplotype frequencies in 1,359 PTC cases and 1,605 controls. *P* values and odds ratios (ORs) are provided using Fisher’s exact test to compare each haplotype with the rest of the haplotypes.

### Image acquisition and statistical analysis

Calculation of percent splicing inclusion was determined using Image Lab v6.0.1 (Bio-Rad). All images and figures were edited in Adobe Photoshop (2017.0.1) and Illustrator (2017.0.2; www.adobe.com), GraphPad Prism (v8.1.2; https://www.graphpad.com), and FlowJo (v10.7)^55^. All graphical data are represented by mean ± SEM and all *P* values reported are calculating from a two-sided Student’s t test. Gene canonical pathway analysis using *EPB41L4A* knockdown was performed using Ingenuity Pathway Analysis software. Gene expression array analysis was performed as previously described^53,54^. Briefly, a filtering method based on percentage of arrays above noise cutoff was applied to filter out low-expression genes. A linear model was employed to detect differentially-expressed genes between conditions. A variance smoothing method with moderated t-statistic was employed. The significance level was adjusted by controlling the mean number of false positives. Statistical software SAS 9.4 and R were used for analysis.

### RT-PCR and qPCR assays

For RNA expression analysis, RNA was extracted using an RNeasy kit, then treated with DNase-I. 500 ng RNA was used for cDNA synthesis using a High-Capacity cDNA Reverse Transcription Kit. PCR of *EPB41L4A-203* splice isoforms was performed using AmpliTaq Gold DNA polymerase under standard PCR conditions. PCR of *EPB41L4A* and *GAPDH* was performed using HotStarTaq DNA polymerase under standard PCR conditions. qPCRs were performed on an ABI Prism 7900 HT Sequence Detection System using either TaqMan gene expression assays (Hs00706279_s1, Hs00223297_m1, 4352934E, and a custom assay for *EPB41L4A-203*) with 2X Universal Fast TaqMan Master Mix or using Fast SYBR Green Master Mix kit under standard conditions, followed by a dissociation stage. For a list of SYBR Green primer pairs see Supplementary Table S1.

### siRNA treatment and microarray hybridization

siRNA treatment and microarray hybridization was performed as previously described^53,54^. Briefly, a non-targeting siRNA pool (Dharmacon) or siRNA pool against *EPB41L4A* (Dharmacon) was transfected into cells using Lipofectamine RNAiMAX. After 72 h, cells were harvested and total RNA was extracted using an RNeasy kit, then treated with DNase-I. The integrity of RNA samples was assessed by BioAnalyzer. Clariom D Human arrays were used to assess gene expression. Totally, 100 ng RNA was used to generate the single-stranded complementary DNA (cDNA) samples for hybridization. Then, cDNA was enzymatically fragmented and biotinylated using the WT Terminal Labeling kit. The cDNA samples were hybridized to the array at 45 °C for 16 h. The arrays were washed and scanned with the Affymetrix GeneChip Scanner 3000 7G system using Affymetrix GeneChip Command Console software. Signal intensities were processed by the robust multiarray average method using Affymetrix Expression Console software.

### Proliferation assay

Equal amounts of the tetrazolium salt, WST-8 ([2-(2-methoxy-4-nitrophenyl)-3-(4-nitrophenyl)-5-(2,4-disulfophneyl)-2H-tetrazolium, monosodium salt)]), were added to BCPAP and NThy-ori 3–1 cells seeded in a 24-well plate transiently transfected over 72 h with non-specific or *EPB41L4A*-specific siRNA. After 72 h, cells and several blank wells filled with medium were treated 1:100 with WST-8 reagent for 1 h. At the end of 1 h, a 100 μl aliquot of supernatant was transferred to a 96-well plate. The absorbance of each well was measured on a NanoDrop One spectrophotometer at 450 nm, normalized to blank wells. Triplicate values were averaged and the experiment was repeated four times.

### Flow cytometry

Following transfection, BCPAP or NThy-ori 3–1 cells were washed with PBS and then resuspended in 500 μl PBS with 5 mM dextrose, and fixed in 1.5 mL ethanol. Fixed cell suspensions were pelleted at 1,500 rpm at 4 °C for 10 min. Ethanol was aspirated and cells were resuspended in 300 μl propidium iodide buffer. Cell suspensions were homogenized using 20 G needles and passed through 100 μm cell strainers into polystyrene tubes. Cell suspensions were then incubated on a shaker in the dark for 15 min and analyzed on a LSR II flow cytometer.

### Ethics approval and consent to participate

Human thyroid cancer specimens were obtained through the Cooperative Human Tissue Network approved by the Office of Responsible Research Practices at The Ohio State University where patients have been appropriately consented.

## Supplementary information

Supplementary Information.

## Data Availability

The datasets generated during and/or analysed during the current study are available in the Gene Expression Omnibus repository with the primary accession code GSE148485.
