# Supplementary material for: Characterizing the function of EPB41L4A in the predisposition to papillary thyroid carcinoma
Source: Sci Rep. 2020 Nov 17;10:19984. doi: 10.1038/s41598-020-76606-0 (PMC7672090; doi:10.1038/s41598-020-76606-0)
Supplement: Supplementary file 1 — Supplementary Information. [file 41598_2020_76606_MOESM1_ESM.docx]

**TITLE**

Characterizing the function of *EPB41L4A* in the predisposition to papillary thyroid carcinoma

**AUTHORS’ NAMES**

Daniel F. Comiskey Jr., Huiling He, Sandya Liyanarachchi, Mehek S. Sheikh, Isabella V. Hendrickson, Lianbo Yu, Pamela L. Brock, and Albert de la Chapelle

**Supplementary Table S1: Primer pairs.**

| Name | Sequence |
| --- | --- |
| rs73227498 F1 EcoRV | aaaaaagatcttgatgagtgctcccaagtcagtg |
| rs73227498 R1 XhoI | gctcgctagcctcgacattttctcgtgggtttgt |
| rs17134155 F1 EcoRV | aaaaaagatcttgatgatttccaagttctctgc |
| rs17134155 R1 XhoI | gctcgctagcctcgaatgtggtctcgattctgta |
| rs17134154 F2 EcoRV | aaaaaagatcttgattggggccatgggtgttgc |
| rs17134154 R2 XhoI | gctcgctagcctcgatcttctcctgcaggctggactacc |
| rs76564228 F2 EcoRV | aaaaaagatcttgatttcccattcttttaccaacataca |
| rs76564228 R2 XhoI | gctcgctagcctcgagagccgtgattgcaccactgt |
| rs56092269 F1 EcoRI | aaaaaagatcttgattgcagtgcctaacatatagtagat |
| rs56092269 R1 XhoI | gctcgctagcctcgataatacagccttcagaaagaaata |
| rs79098204 F1 EcoRV | aaaaaagatcttgatgtcctagaaatctgccaactt |
| rs79098204 R1 XhoI | gctcgctagcctcgaatgactaactgccctgaaactgag |
| rs73787778 F1 EcoRV | aaaaaagatcttgatcctctccttcgaatcccacg |
| rs73787778 R1 XhoI | gctcgctagcctcgaaaatggtgcttacaggaacttg |
| rs76177172 F2 EcoRV | aaaaaagatcttgatgtcaatgaatttctccactgct |
| rs76177172 R2 XhoI | gctcgctagcctcgaaaagatctatcacaacgaactgc |
| rs27981 F2 EcoRV | aaaaaagatcttgataaacactggtcattctctactg |
| rs27981 R2 XhoI | tcgctagcctcgacctctgccctgtcccttcc |
| ANTXR2 F2 | gctttgtcctgcacctatcct |
| ANTXR2 R2 | gggccaaaaccaccacatcaaacc |
| ARHGAP19 F1 | accaggaggagacccagcaccata |
| ARHGAP19 R1 | agcgcgaacgagccctcttttgta |
| BRAF F1 | aatttttatggtgggacgaggat |
| BRAF R1 | ccgattcaaggagggttctg |
| CCDC6 F4 | agttgagacgggagaagattgacc |
| CCDC6 R4 | ctaggcgatggtggagcagaga |
| CDH6 F4 | ttaccgctacttcttgctgctctt |
| CDH6 R4 | cctcttggtggcctgtatgtcg |
| CTNNB1 F2 | gcgtggacaatggctactca |
| CTNNB1 R2 | aaaatccctgttcccactcatac |
| DICER1 F1 | cacaaaattggcgaactggatgac |
| DICER1 R1 | cttttgggtagcactgccttcgtt |
| DKK2 F5 | ggtactcggcacagagatcg |
| DKK2 R5 | ccctgatggagcactggttt |
| DNMT3B F2 | gcacccaagcgcctcaagac |
| DNMT3B R2 | acacggggtttttcctgccacaag |
| EGFR F2 | caccgtggcttgcattgatagaa |
| EGFR R2 | cagcgggccttttgggaacg |
| EPB41L4A-201 F2 | atctgagtaccggtttgttcctga |
| EPB41L4A-201 R2 | ttccccacttgctttttattcttg |
| EPB41L4A-AS1-201 F1 | gaagatggatgcctgaagtgtaga |
| EPB41L4A-AS1-201 R1 | ctccagcaggtaattatgtcagta |
| EPB41L4A-203 X11 F2 | tgctacagttcatcaggtatgtg |
| EPB41L4A-203 Cloning R2 | gtgttggcctcattcttactattg |
| EPB41L4A-203 X8 F1 | agccccgatatccaagcaaaag |
| EPB41L4A-203 X11-13 R2 | tggacaccctgatgaactg |
| EPB41L4A-203 X11-14 F1 | acagttcatcagtgcagactct |
| GAPDH F1 | ggcatggactgtggtcatgag |
| GAPDH R1 | tcttctgggtggcagtgatg |
| HIPK3 F1 | tgcagccactacagatccgaccag |
| HIPK3 R2 | aaatcatcttcccccagtctcca |
| IGFBP6 F4 | gtgaaccgcagagaccaacagag |
| IGFBP6 R4 | agcccctcggtagacctcagt |
| LIPH F2 | aaggagccattaggaaacatagac |
| LIPH R2 | acagggatacgcagtgatgg |
| LPCAT2 F1 | gcgaggcgggtccagattgtc |
| LPCAT2 R1 | attgggtgggtcagcttttcagga |
| MAP2 F1 | ccaggtggcggacgtgtgaaaa |
| MAP2 R1 | tctgcctggggactgtgtaatgat |
| MAPKAPK3 F2 | agccataacattgcccaccgagat |
| MAPKAPK3 R2 | tcaggggccacataatagggagta |
| NRG1 F4 | atctgcatcgccctccttgtggtc |
| NRG1 R4 | gggggtgggttaggatggtgagg |
| PLEKKH1 F1 | cgccatgcagatcccagaagtgaa |
| PLEKKH1 R1 | aggggttacgcagcaggatggaca |
| RAF1 F4 | ctcggattgggtcaggctcttttg |
| RAF1 R2 | tttgcgcagaacagccacctcatt |
| RPS6KA5 F1 | tgccctcgaacatctccacaag |
| RPS6KA5 R1 | tcctgaatctccccctctgacaat |
| S1PR1 F5 | ggtgacttcgcgtagcagg |
| S1PR1 R5 | cgactgactgcgtagtgctc |
| SNAI1 F6 | ctaggccctggctgctacaa |
| SNAI1 R6 | ccggactcttggtgcttgtg |
| STC1 F1 | atgaggcggagcagaatgact |
| STC1 R1 | gcagcgctgtacaagaaggat |
| TNFSF15 F7 | aaaggacaggagtttgcacct |
| TNFSF15 R7 | actctgggatcagcaggaat |

**Supplementary Table S2: Linkage Disequilibrium (LD) Analysis of 5q22**

| Chr | Position | dbSNP | AlleleA | AlleleB | D' | r^2^ | GWAS  Log_10_  P value | GTEX  P value | RegulomeDB | DANN Score |
| --- | --- | --- | --- | --- | --- | --- | --- | --- | --- | --- |
| 5 | 111390730 | rs115712814 | C | G | 0.84 | 0.41 | 3.39 |  |  |  |
| 5 | 111392018 | rs76675783 | G | A | 0.85 | 0.42 | 2.78 | 6.55E-03 | 7 | 0.61 |
| 5 | 111401336 | rs199653421 | G | GCA | 0.86 | 0.41 | 2.79 |  |  |  |
| 5 | 111401338 | rs30295 | G | A | 0.76 | 0.37 | 2.59 |  |  | 0.72 |
| 5 | 111432540 | rs27413 | C | T | 0.84 | 0.33 | 3.40 | 2.19E-02 | 7 | 0.71 |
| 5 | 111455860 | rs191762127 | T | C | 1.00 | 0.00 | 2.31 |  | 3a | 0.51 |
| 5 | 111456583 | rs78788069 | C | T | 0.96 | 0.83 | 5.14 | 7.04E-06 | 5 | 0.64 |
| 5 | 111462440 | rs73227487 | A | G | 0.87 | 0.69 | 4.04 | 5.41E-04 | 7 | 0.56 |
| 5 | 111463837 | rs27982 | T | C | 0.97 | 0.78 | 4.39 | 3.55E-04 | 2c | 0.36 |
| 5 | 111465432 | rs27342 | T | C | 0.99 | 0.44 | 2.37 | 9.80E-03 | 6 | 0.84 |
| 5 | 111465546 | rs17253030 | C | G | 0.88 | 0.71 | 3.94 | 2.18E-04 | 5 | 0.79 |
| **5** | **111465851** | **rs27981** | **G** | **A** | **0.99** | **0.81** | **4.19** | **7.03E-04** | **6** | **0.79** |
| 5 | 111466355 | rs25788 | T | C | 0.99 | 0.81 | 4.04 | 7.83E-05 | 7 | 0.48 |
| 5 | 111467623 | rs200001835 | A | AAAAAAT | 0.99 | 0.82 | 4.49 |  |  |  |
| 5 | 111467814 | rs6594564 | T | C | 0.99 | 0.81 | 4.19 | 2.72E-04 | 6 | 0.58 |
| 5 | 111468119 | rs138459527 | C | A | 0.88 | 0.71 | 3.94 |  |  |  |
| 5 | 111468607 | rs148615021 | T | A | 0.99 | 0.89 | 4.45 | 1.15E-05 | 6 | 0.46 |
| 5 | 111469880 | rs6863562 | G | A | 0.99 | 0.56 | 4.14 |  | 5 | 0.22 |
| 5 | 111470230 | rs7728316 | T | C | 0.99 | 0.81 | 3.87 |  | 7 | 0.41 |
| 5 | 111471712 | rs73787773 | T | C | 0.88 | 0.78 | 5.08 | 1.06E-04 | 6 | 0.77 |
| 5 | 111472395 | rs58079319 | G | A | 0.88 | 0.78 | 4.91 | 1.39E-05 | 7 | 0.77 |
| 5 | 111472541 | rs57673629 | A | G | 0.99 | 0.88 | 5.05 | 2.47E-05 | 5 | 0.38 |
| 5 | 111472611 | rs73787775 | C | A | 0.88 | 0.78 | 5.10 | 1.01E-04 | 4 | 0.44 |
| 5 | 111473340 | rs73227490 | T | C | 0.99 | 0.89 | 5.24 | 4.77E-05 | 6 | 0.67 |
| 5 | 111473369 | rs73227492 | A | G | 0.88 | 0.78 | 5.03 | 2.61E-05 | 7 | 0.61 |
| 5 | 111473440 | rs6866157 | T | G | 0.99 | 0.47 | 3.06 | 1.05E-02 | 7 | 0.70 |
| 5 | 111473661 | rs113965051 | G | A | 1.00 | 0.52 | 3.97 | 1.12E-05 | 7 | 0.47 |
| **5** | **111474244** | **rs76177172** | **C** | **T** | **0.99** | **0.98** | **5.63** | **2.29E-06** | **5** | **0.77** |
| 5 | 111476097 | rs78790140 | T | C | 0.99 | 0.98 | 5.63 | 1.41E-06 | 6 | 0.43 |
| 5 | 111477659 | rs73227493 | A | G | 1.00 | 0.89 | 5.62 | 1.08E-06 | 5 | 0.32 |
| **5** | **111477958** | **rs73787778** | **G** | **C** | **0.88** | **0.78** | **5.10** | **2.72E-05** | **5** | **0.81** |
| 5 | 111478211 | rs55641410 | C | T | 0.99 | 0.98 | 5.65 | 2.55E-06 | 7 | 0.62 |
| **5** | **111478820** | **rs79092804** | **C** | **T** | **1.00** | **0.89** | **5.75** | **1.54E-06** | **2b** | **0.58** |
| **5** | **111478829** | **rs79705195** | **G** | **A** | **0.99** | **0.98** | **5.65** | **2.29E-06** | **4** | **0.72** |
| **5** | **111478870** | **rs78455089** | **C** | **A** | **1.00** | **0.99** | **5.78** | **3.36E-06** | **4** | **0.66** |
| **5** | **111479053** | **rs56092269** | **T** | **G** | **0.99** | **0.88** | **5.15** | **1.59E-05** | **2b** | **0.61** |
| 5 | 111479794 | rs57349429 | A | T | 0.99 | 0.88 | 5.48 | 2.35E-05 | 5 | 0.49 |
| 5 | 111479955 | rs17134152 | G | T | 0.99 | 0.88 | 5.27 | 1.59E-05 | 7 | 0.39 |
| **5** | **111479971** | **rs76564228** | **A** | **G** | **0.99** | **0.98** | **5.65** | **2.29E-06** | **5** | **0.72** |
| 5 | 111480324 | rs17134153 | C | T | 0.99 | 0.98 | 5.65 | 2.29E-06 | 6 | 0.49 |
| **5** | **111480534** | **rs17134154** | **T** | **C** | **0.99** | **0.98** | **5.56** | **2.29E-06** | **3a** | **0.77** |
| 5 | 111480610 | rs73787779 | G | C | 0.99 | 0.88 | 5.27 | 1.05E-05 | 3a | 0.61 |
| 5 | 111480685 | rs73227496 | A | T | 0.88 | 0.78 | 5.25 | 2.17E-05 | 4 | 0.20 |
| 5 | 111480859 | rs112564823 | A | G | 0.88 | 0.78 | 5.25 | 1.80E-05 | 5 | 0.34 |
| 5 | 111481403 | rs56010932 | A | C | 0.99 | 0.88 | 5.30 | 1.59E-05 | 6 | 0.52 |
| 5 | 111481600 | rs56071312 | A | T | 0.99 | 0.88 | 5.34 | 1.48E-05 | 5 | 0.65 |
| **5** | **111481696** | **rs17134155** | **C** | **T** | **0.99** | **0.67** | **5.53** | **6.95E-04** | **3a** | **0.58** |
| 5 | 111481737 | rs17134156 | A | T | 0.99 | 0.98 | 5.72 | 2.29E-06 | 4 | 0.27 |
| 5 | 111484212 | rs60370278 | T | G | 0.88 | 0.78 | 5.28 | 6.31E-05 | 6 | 0.75 |
| 5 | 111485084 | rs57541807 | G | A | 0.89 | 0.79 | 5.30 | 2.81E-05 | 6 | 0.76 |
| **5** | **111485904** | **rs73227498** | **A** | **T** | **1.00** | **1.00** | **5.86** | **2.56E-06** | **3a** | **0.57** |


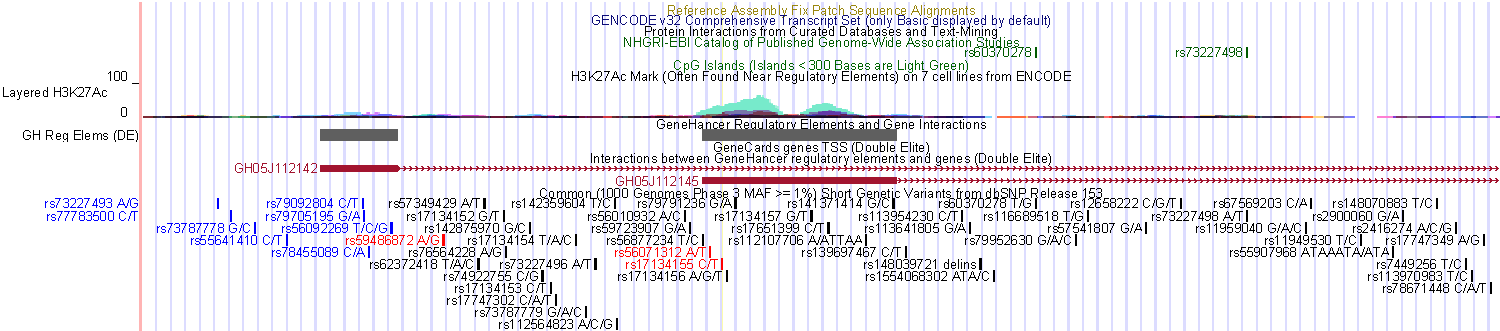


**Supplementary Figure S1: rs17134155 lies within a GeneHancer regulatory element.** University of Santa Cruz Genome Browser view at 5q22 with tracks depicting (top to bottom): a catalog of published GWAS variants, layered H3K27Ac in 7 cell lines from ENCODE, GeneHancer regulatory elements, and common SNPs from 1000 Genomes Phase 3 data. GeneHancer regulatory elements are depicted as grey boxes; the position of GWAS SNP rs73227498 is shown above in green and the position of rs17134155 is shown below in red.


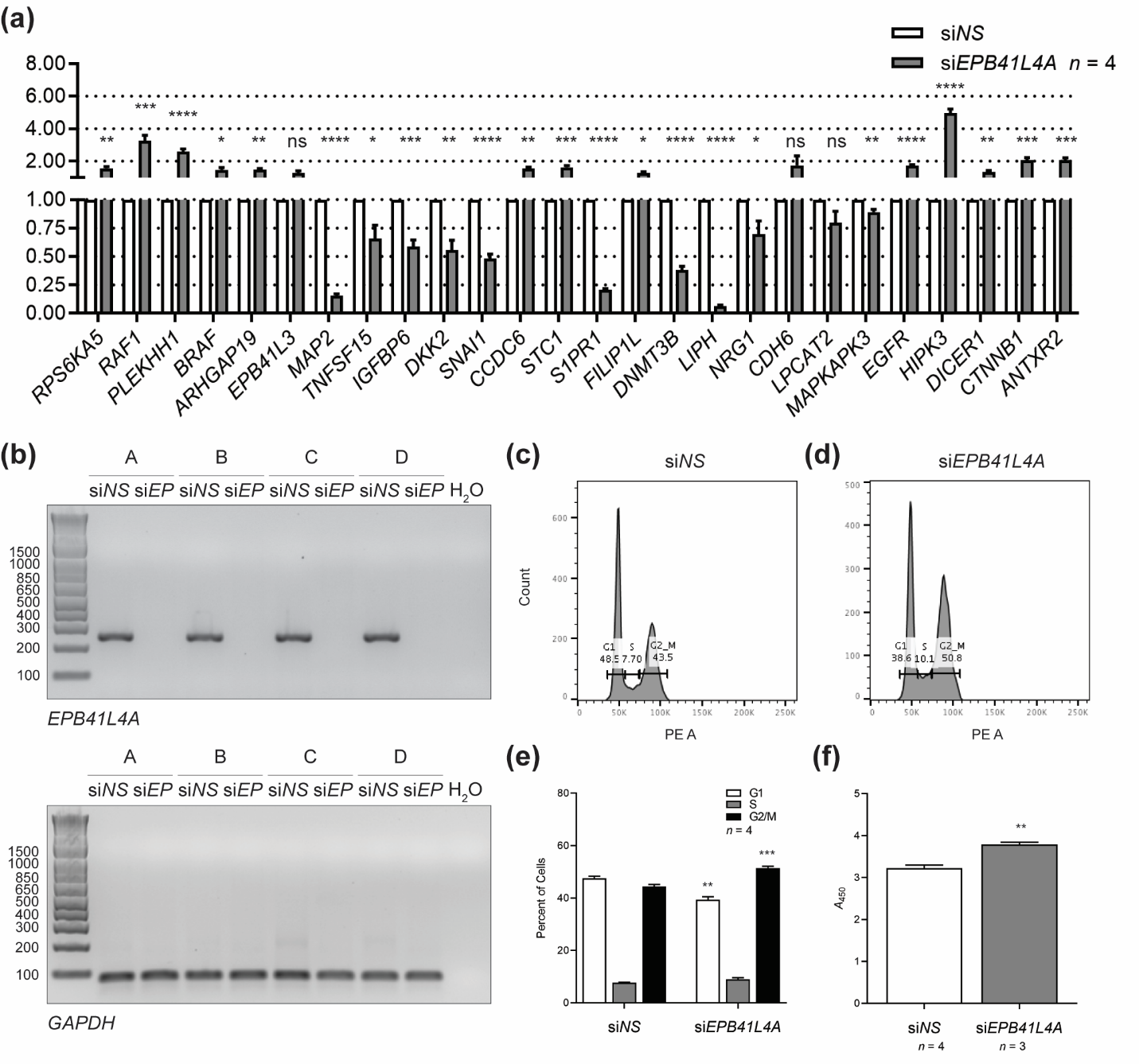


**Supplementary Figure 2: Knockdown of *EPB41L4A* results in increased proliferation of NThy-ori 3-1 cells.** Quantitative polymerase chain reaction (qPCR) validations for twenty-six differentially-expressed genes in NThy-ori 3-1 cells treated with non-specific (si*NS*) or *EPB41L4A* (si*EPB41L4A*) siRNA. **b)** RT-PCR of *EPB41L4A* (top panel) and *GAPDH* (bottom panel) expression in NThy-ori 3-1 cells treated with si*NS* or si*EPB41L4A* (si*EP*), *n* = 4. Histogram of propidium iodide-stained NThy-ori 3-1 cells treated with **c)** si*NS* or **d)** si*EPB41L4A*. The percentage of cells in each phase of the cell cycle (G1, S, G2/M) is shown for each range. **e)** The average percentage of NThy-ori 3-1 cells in each phase of the cell cycle is shown. **f)** WST-8 proliferation assay of NThy-ori 3-1 cells. The level of metabolic activity is depicted by the absorbance of formazan dye (*A* = 450). ns (not significant), **P* < 0.05, ***P* < 0.01, ****P* < 0.001, *****P* < 0.0001 (Two tailed t-test).
